# Analysis of Ni, Cu, and Fe in Loose Cosmetic Products: A Preliminary Dermal Exposure and Health Risk Assessment

**DOI:** 10.3390/toxics14090766

**Published:** 2026-08-27

**Authors:** Agnieszka Berdowska, Katarzyna Bandurska, Joanna Kończyk, Iwona Zawierucha

**Affiliations:** Faculty of Science and Technology, Jan Dlugosz University, 42-200 Czestochowa, Poland; a.berdowska@ujd.edu.pl (A.B.); j.konczyk@ujd.edu.pl (J.K.); i.zawierucha@ujd.edu.pl (I.Z.)

**Keywords:** heavy metals, nickel, copper, iron, loose cosmetics, microwave plasma–atomic emission spectrometry, chronic daily dermal exposure, lifetime cancer risk, dermal exposure

## Abstract

Loose cosmetic products are widely used in everyday makeup application; however, they may constitute a source of consumer exposure to metals present either intentionally as structural mineral pigments or unintentionally as contaminants derived from raw materials. The aim of this study was to quantitatively determine the concentrations of nickel, copper, and iron in loose facial cosmetics (including face powders, contouring products, and eyeshadows) available on the Polish market and to evaluate the potential human health risks. Following validated microwave digestion, the samples were analyzed using microwave plasma–atomic emission spectrometry (MP-AES). Non-carcinogenic and carcinogenic health risks were assessed for dermal exposure by calculating the Hazard Quotient (HQ), Hazard Index (HI), and lifetime cancer risk (LCR). The analyzed metals were detected in the majority of the tested products. Nickel was not detected in four cosmetic samples, whereas its concentration in the remaining products ranged from 0.45 to 37.57 mg/kg. Copper concentrations were between 0.83 and 35.39 mg/kg, while iron concentrations varied from 0.03 × 10^3^ to 109.56 × 10^3^ mg/kg. The calculated HQ and HI values (maximum HI = 8.91 × 10^−5^) were well below 1, indicating no significant non-carcinogenic health risk. The estimated lifetime cancer risk for nickel was within the safe range (5.63 × 10^−12^ to 8.28 × 10^−9^). Chronic dermal exposure to the analyzed metals is therefore unlikely to pose either systemic toxic or carcinogenic risks to consumers. However, the detected levels of nickel and copper in the samples with the highest concentrations may trigger local allergic reactions in sensitized individuals. These findings highlight the importance of monitoring the purity of mineral raw materials used in cosmetic production.

## 1. Introduction

Cosmetics are an integral part of daily consumer routines, with historical roots tracing back to ancient civilizations where formulations utilized plant- and animal-derived raw materials [1]. Modern cosmetic products frequently contain heavy metals, introduced either intentionally as functional components and mineral pigments, or unintentionally as raw-material contaminants derived from insufficient purification or manufacturing deficiencies [2,3]. Although trace concentrations of these elements generally pose no immediate systemic health risk after a single application, long-term chronic use of contaminated products can lead to bioaccumulation and adverse local cutaneous reactions, such as contact dermatitis or urticaria [4]. Consequently, strict legislative frameworks prohibit or rigorously restrict the permissible concentrations of specific substances to safeguard public health [5]. Therefore, when evaluating metal concentrations in decorative formulations, a strict toxicological distinction must be made between elements intentionally incorporated for structural or pigmentary functionality, such as iron in iron oxides, and genuine, non-functional chemical impurities like nickel. Decorative facial makeup items enhance physical appearance and emphasize facial features, though they rarely provide significant skincare benefits and typically contain complex colorant or fragrance mixtures [6]. Individual skin responses to these chemical components vary considerably, necessitating careful consumer adherence to product instruction guidelines and ingredient list checks prior to application [7]. Loose cosmetics constitute a distinct category defined by powdered raw materials applied to the skin via synthetic or natural bristle brushes and cosmetic puffs [1,8]. These products are routinely applied either directly onto cleansed skin or layered over wet liquid foundations or cosmetic makeup primers to enhance surface adhesion and longevity [9]. Face powder is predominantly utilized to set makeup bases, whereas loose blushes, bronzers, and highlighters define facial contours and introduce specialized shade variations across different facial regions. Eyeshadows are designed specifically to enhance the ocular area, presenting a broad spectrum of color shades [10]. Heavy metal absorption into the human body occurs via three distinct physiological routes: dermal absorption, ingestion, and inhalation [11].

According to European Regulation (EC) No 1223/2009, nickel and all its compounds are completely prohibited as intentionally added ingredients in cosmetic products [5]. The only legal exception concerns trace amounts that are technically unavoidable and arise under good manufacturing practice, for example, as impurities in natural mineral pigments. In contrast, the regulatory approach in the United States is more permissive than in Europe. The U.S. Food and Drug Administration (FDA) has not imposed a specific ban on nickel in cosmetics [12]. However, it regulates its presence indirectly through strict purity requirements for color additives and mineral pigments (e.g., iron oxides), which are among the most common sources of nickel contamination [13].

Nickel allergy is a relatively common health problem. The most frequent sensitizing compounds of this element are its salts [14]. Repeated exposure may lead to the accumulation of nickel in the skin and may result in allergic contact dermatitis [13].

Copper is considered an element in cosmetology that may contribute to the activation and acceleration of skin tissue regeneration processes. It also exhibits antimicrobial properties, which have been utilized since antiquity for the cleansing and treatment of skin lesions [15]. Although metallic copper is associated with a low allergenic potential, some of its derivatives exhibit strong irritant effects. A particular risk is associated with the application of copper(II) sulfate. The use of copper-containing preparations on a damaged epidermal barrier may induce local toxic and allergic reactions. The most frequently reported clinical symptoms include severe skin irritation, acute inflammation, and intense pruritus [16].

Copper may become a potential irritant when it undergoes oxidation, leading to the formation of free acids. The oxidative activity of these acids may subsequently trigger skin irritation upon reaching the viable layers of the skin. Copper may also interact with reactive oxygen species, resulting in the formation of hydroxyl radicals that inactivate enzymes and damage cell membranes and organelles [16].

Iron supports skin regeneration in the dermis, detoxification processes, and collagen synthesis. In cosmetic products, it plays a regenerative and skin-moisturizing role [17]. Iron and its compounds are used not only for their skincare functions but also, in color cosmetics, as pigments. Iron oxides are common ingredients in facial cosmetics. Due to their excellent covering properties even at low concentrations, they provide intense coloration and contribute to a matte skin appearance. Owing to their protective properties, they are also used in sunscreens [18]. Iron oxides occur in three main color forms: red (iron(III) oxide/CI 77,491), black (iron(II) oxide/CI 77,489 or the related CI 77,499), and yellow (hydrated iron(III) oxide/CI 77,492) [19].

Although iron oxides are generally recognized as safe colorants, their presence in facial cosmetic products may be associated with certain health risks. The most common dermatological concern is the induction of local allergic reactions, including contact dermatitis. Moreover, prolonged use of products contaminated with this metal may pose a risk of bioaccumulation, which in extreme cases may increase oxidative stress or lead to cellular toxicity [4].

The aim of this study was to quantitatively assess the content of selected metals (nickel, copper, and iron) in loose cosmetic products intended for face and eye makeup (face powders, contouring products, and eyeshadows) available on the Polish retail market. An important aspect of the study was the evaluation of potential health risks associated with their long-term application. The novelty of the research lies in the fact that, although the presence of metals in liquid and emulsion-based cosmetic products has been extensively investigated [20,21,22,23,24], loose cosmetic formulations remain relatively underrepresented in the recent toxicological literature, particularly in the national context.

The choice of the three specific elements investigated in this work—nickel, copper, and iron—was dictated by their distinct technological functions and toxicological significance in loose cosmetic matrices. Iron represents a structural and pigmentary backbone of decorative cosmetics (primarily via iron oxides), whereas nickel is a strictly prohibited substance under European law and a potent contact allergen [5]. Copper occupies an intermediate position, acting occasionally as a functional agent but also posing risks related to local skin irritation and the generation of reactive oxygen species. Investigating this triad allows for a balanced evaluation of intentional ingredient quality versus unintentional contamination.

Although the occurrence of metal contaminants in certain loose cosmetic products (especially eyeshadows) has been previously reported [23,25,26,27,28], this study provides added value through an integrated comparative assessment of three distinct categories of loose products used on different facial areas. The obtained results provide a preliminary screening-level insight into the performance of European quality control and safety assessment procedures for loose cosmetic products under real conditions of the Polish retail market, highlighting the practical importance of independent pilot monitoring.

## 2. Materials and Methods

### 2.1. Sample Collection and Preparation

The study included three groups of loose cosmetic products intended for face and eye makeup: face powders (coded as P1–P4), contouring products (bronzer (BR1), blush (BL1), and highlighters (H1–H2)), and eyeshadows (E1–E6). All cosmetic products analyzed in this study were purchased on the Polish market. The country of origin was not considered during the selection of cosmetic products. To ensure an initial screening across different sectors, the study applied a stratified random sampling strategy within a limited exploratory sample. Specifically, the cosmetic products were chosen to cover different commercial segments and retail price tiers, including budget (low-cost), mass-market (medium), and premium formulations. All products were purchased from major Polish retail pharmacies, drugstores, and leading cosmetics chains to focus the preliminary screening on widely available consumer brands. The country of origin and specific batch numbers were recorded for internal tracking but were not considered as independent grouping variables during the initial selection. After purchase, the products were stored in their original, sealed commercial packaging at controlled room temperature (20 ± 2 °C) and away from light. To prevent any external contamination, the containers were opened, dried to a constant weight, and mechanically homogenized immediately prior to the microwave digestion and instrumental analysis. The INCI compositions of all analyzed products are provided in the Supplementary Materials (Table S1).

### 2.2. Reagents and Standard Solutions

For microwave digestion of the cosmetic samples, 65% nitric acid (HNO_3_, Sigma-Aldrich, St. Louis, MO, USA) and ≥30% hydrogen peroxide (H_2_O_2_, Supelco, Bellefonte, PA, USA), both of analytical grade and suitable for inorganic trace analysis, were used. Quantitative analysis and validation of the analytical procedure were performed using certified reference standards (CPAChem, Bogomilovo, Bulgaria) consisting of single-element standard solutions of Ni, Cu, and Fe at a concentration of 1000 mg/L.

### 2.3. Microwave Digestion and MP-AES Analysis

Approximately 0.2000 g of each homogenized cosmetic sample was weighed and transferred into Teflon digestion vessels. A mixture of 5 mL of 65% HNO_3_ and 1 mL of 30% H_2_O_2_ was then added. The vessels were sealed and subjected to microwave digestion using a Topex+ system (Preekem, Shanghai, China) under a three-step program: 3 min at 120 °C, 3 min at 180 °C, and 15 min at 200 °C, with 8 min ramps between steps. Temperature and pressure were automatically monitored in a dedicated control vessel throughout each digestion run. During the final digestion step, the pressure reached a maximum of 17.6 bar, remaining well below the maximum permissible operating pressure of 50 bar. After digestion, the vessels were cooled in the instrument to 50 °C for approximately 25–30 min before opening. The digests were diluted with deionized water and filtered through 0.45 µm syringe filters before instrumental analysis. For samples in which visible residues remained after the initial microwave-assisted digestion, the residues were subjected to a second digestion cycle under the same conditions.

Metal concentrations were determined using a microwave plasma atomic emission spectrometer (MP-AES 4210, Agilent Technologies, Santa Clara, CA, USA) under the operating conditions presented in Table 1.

The metal concentrations in the cosmetic samples were calculated based on the sample mass, the final digest volume, and the results of spectrometric analysis of three independently prepared portions of each cosmetic sample.

### 2.4. Quality Control and Method Validation

To confirm the suitability of the applied analytical procedure for determining Fe, Ni, and Cu in color cosmetics, selected validation parameters were evaluated. Linearity was assessed based on the coefficients of determination obtained for calibration curves across the investigated concentration ranges. The limits of detection (LOD) and limits of quantification (LOQ) were determined as three and nine times, respectively, the standard deviation (SD) obtained from seven replicate measurements of the lowest calibration standard. Repeatability and intermediate precision were evaluated through the analysis of procedural blanks, calibration standards, and replicate sample measurements conducted within a single day and over three consecutive days. The accuracy of the analytical procedure was further assessed by spike-recovery experiments, in which known amounts of Ni, Cu, and Fe were added to cosmetic samples prior to microwave digestion.

Given the high Fe concentrations in some analyzed samples, the potential effect of Fe on Ni and Cu determination was investigated at three concentration levels, 15, 90, and 210 mg/L, corresponding approximately to the Fe concentrations in the digests of samples P1, P4, and BR1, respectively. Changes in Ni and Cu emission signals following Fe addition, as well as differences in the slopes of calibration curves prepared in the absence and presence of Fe, were compared.

Digestion completeness was verified by re-digesting the residues remaining after the initial digestion and determining Fe, Ni, and Cu in the resulting solutions. The impact of alterations in digestion conditions (robustness) was assessed by implementing three modifications to the procedure: excluding the addition of H_2_O_2_, prolonging the duration of the final program step to 20 min, and raising the temperature of the final step to 210 °C.

### 2.5. Health Risk Assessment

The chronic daily dermal exposure (CDDE) was calculated using the following equation [29,30,31]:

(1) $$CDDE=\frac{CS\times SA\times AF\times ABS\times EF\times ED\times CF}{BW\times AT},$$

where

CS—the concentration of toxic elements in cosmetics (mg/kg);

SA—exposed skin area (cm^2^) (for powders: 565 cm^2^ according to the SCCS Notes of Guidance [32]; for blush and bronzer: 300 cm^2^ in accordance with the Dutch National Institute for Public Health and the Environment (RIVM) [33]; for highlighters: 200 cm^2^, adopted as a conservative screening-level dermal exposure estimate adapted from the localized application metrics of the SCCS guidelines [32] due to the absence of specific statutory thresholds; for eyeshadows: 24 cm^2^ according to the SCCS Notes of Guidance [32]).

AF—adherence factor (mg/cm^2^) (0.07 mg/cm^2^) [31,34];

ABS—dermal absorption fraction (no units) (0.001) [35];

EF—exposure frequency (days/year) (that is 365 days per year) [36];

ED—exposure duration (year) (represents exposure duration for an adult consumer as 30 years) [35];

CF—units conversion factor (kg/mg) (10^−6^);

BW—body weight (kg) (the adult body weight as 70 kg) [35];

AT—averaging time (days) (calculated as EF × ED (i.e., 10,950 days) for non-carcinogenic risks, and fixed at 70 years × 365 days/year (i.e., 25,550 days) for carcinogenic risks) [34,36].

To ensure maximum consumer safety and account for exposure uncertainties, the mathematical model was intentionally parameterized under the worst-case scenario principle. Specifically, the exposure calculations assume direct application of the loose cosmetic formulations to bare skin, completely omitting any potential mitigating or barrier effects that a previously applied wet liquid foundation or makeup primer might provide.

The Hazard Quotient (HQ) is the ratio of exposure to hazardous substances to the chronic reference dose (RfD_d_) of the toxicant and was calculated using the following formula [36]:

(2) $$HQ=\frac{CDDE}{{RfD}_{d}},$$

where

RfD_d_—dermal reference dose (mg·kg^−1^·day^−1^) (for Ni: 5.4 × 10^−3^ mg·kg^−1^·day^−1^ [23,37,38], for Cu: 4.0 × 10^−2^ mg·kg^−1^·day^−1^ [37,39] and for Fe: 0.7 mg·kg^−1^·day^−1^ [37,39]). HQ values ≤ 1 reflect safe exposure levels without significant health risks, while values exceeding unity (>1) surpass safety thresholds and indicate potential toxicity [23].

The Hazard Index (HI) was calculated as the sum of individual HQ values to assess the cumulative risk of multi-element exposure [39]:

(3) $$HI=\sum_{i=1}^{n}{HQ}_{i}$$

where

n—number of different elements that are present in a given cosmetic.

An HI > 1 signals that the combined exposure exceeds safe thresholds [23].

The lifetime cancer risk (LCR) for carcinogenic heavy metals (Ni) was determined using the following formula:

LCR = CDDE × SF,(4)

where

SF—carcinogenicity slope factor ((mg·kg^−1^·day^−1^)^−1^) (for Ni 0.91 (mg·kg^−1^·day^−1^)^−1^) [22,23]. For heavy metals, an LCR below 1 × 10^−6^ is considered negligible, and the cancer risk can be disregarded, whereas an LCR above 1 × 10^−4^ is considered detrimental and problematic. Values falling within the range of 1 × 10^−6^ to 1 × 10^−4^ are commonly utilized as a risk assessment benchmark, indicating a level of exposure that is generally considered acceptable or tolerable in toxicological evaluations [25,40].

### 2.6. Statistical Analysis

All experimental analyses were performed in triplicate, and the chemical concentration data were presented as mean values ± standard deviation (SD). To ensure a comprehensive evaluation, two separate one-way analyses of variance (ANOVA) followed by Tukey’s post hoc honest significant difference (HSD) test were executed. First, to perform pairwise comparisons among individual cosmetic products, the 14 commercial products were defined as independent categorical groups, and the three independent analytical replicates per product were utilized as separate observations (*n* = 3) per group, totaling 42 observations. This step was applied to verify whether the inter-formulation compositional variations significantly exceeded the internal analytical variance introduced during sample preparation, microwave digestion, and MP-AES measurement. Second, to evaluate significant differences among the overarching functional product categories (face powders, contouring products, and eyeshadows), a separate one-way ANOVA was performed. To prevent pseudoreplication in this category-level assessment, the independent commercial product was defined as the statistical unit (*n* = 14), and the analysis was executed using the mean concentrations of each product. Potential global linear relationships and shared manufacturing origins among nickel, copper, and iron concentrations were investigated across the entire dataset by calculating Pearson correlation coefficients (r). To avoid pseudoreplication and ensure statistical independence, this global correlation analysis was based strictly on one mean value per independent commercial product (*n* = 14). Due to the limited number of products within the individual functional categories (*n* = 4 for face powders, *n* = 4 for contouring products, and *n* = 6 for eyeshadows), category-specific correlation analyses were omitted to prevent low statistical power. Advanced multivariate statistical techniques were applied to uncover deeper structural similarities and chemical stratification across the commercial products. For the execution of multivariate models, values below the limit of detection for nickel were handled using the standard substitution method, where they were replaced with a value equal to half of the detection limit (LOD/2, i.e., 0.0665 mg/kg). Principal component analysis (PCA) was conducted based on the standardized correlation matrix to extract eigenvalues, factor loadings, and factor scores, projecting multi-element variance onto a two-dimensional biplot. Complementarily, hierarchical cluster analysis (HCA) was performed on the standardized mean dataset using Ward’s minimum variance method as the agglomerative linkage criterion and Euclidean distance as the proximity measure. To accommodate the substantial order-of-magnitude variation across matrix components without losing micro-compositional resolution, a log-transformation was applied to the linkage distance axis in the final topological dendrogram. Statistical evaluation was executed using Statistica software (version 14, TIBCO Software Inc., Palo Alto, CA, USA). Statistical significance for all tests was established at *p* < 0.05.

## 3. Results

### 3.1. Analytical Performance and Validation Metrics

To ensure the reliability of the analytical results, the microwave-assisted digestion procedure and subsequent MP-AES determination of Fe, Ni, and Cu were validated in terms of linearity, sensitivity, precision, accuracy, matrix effects, digestion completeness, and robustness. The main validation parameters are presented in Table 2.

Excellent linearity was obtained for all analytes (R^2^ > 0.9999). The LODs and LOQs were suitable for the analysis of cosmetic samples; only Ni concentrations in samples H2, E2, E3, and E6 were below the LOD. The obtained precision values, expressed as relative standard deviation (RSD), demonstrated good repeatability of the method, with individual values ranging from 2.6 to 9.1%, and satisfactory stability of results obtained on different analytical days, as reflected by intermediate precision, with individual values ranging from 1.8% to 11.4%. Greater variability was observed for Ni and Cu than for Fe, which may be attributed to their lower concentrations in the samples and to the greater influence of heterogeneity in the complex cosmetic matrix. Spike recoveries in the individual cosmetic samples ranged from 96.0% to 98.2% for Ni, 92.8% to 96.8% for Cu, and 94.7% to 97.5% for Fe, confirming satisfactory method accuracy. Matrix effects related to high Fe concentrations were observed only for low Ni and Cu concentrations; therefore, dilution or standard-addition calibration was used where necessary. The applied digestion procedure was robust to minor variations in digestion time and temperature, whereas the presence of H_2_O_2_ was essential for efficient decomposition of the cosmetic matrix. Detailed results of the matrix-effect digestion-completeness and robustness studies are provided in the Supplementary Materials (Figure S1).

### 3.2. Concentrations of the Analyzed Metals in Cosmetic Samples

**Figure 1:**
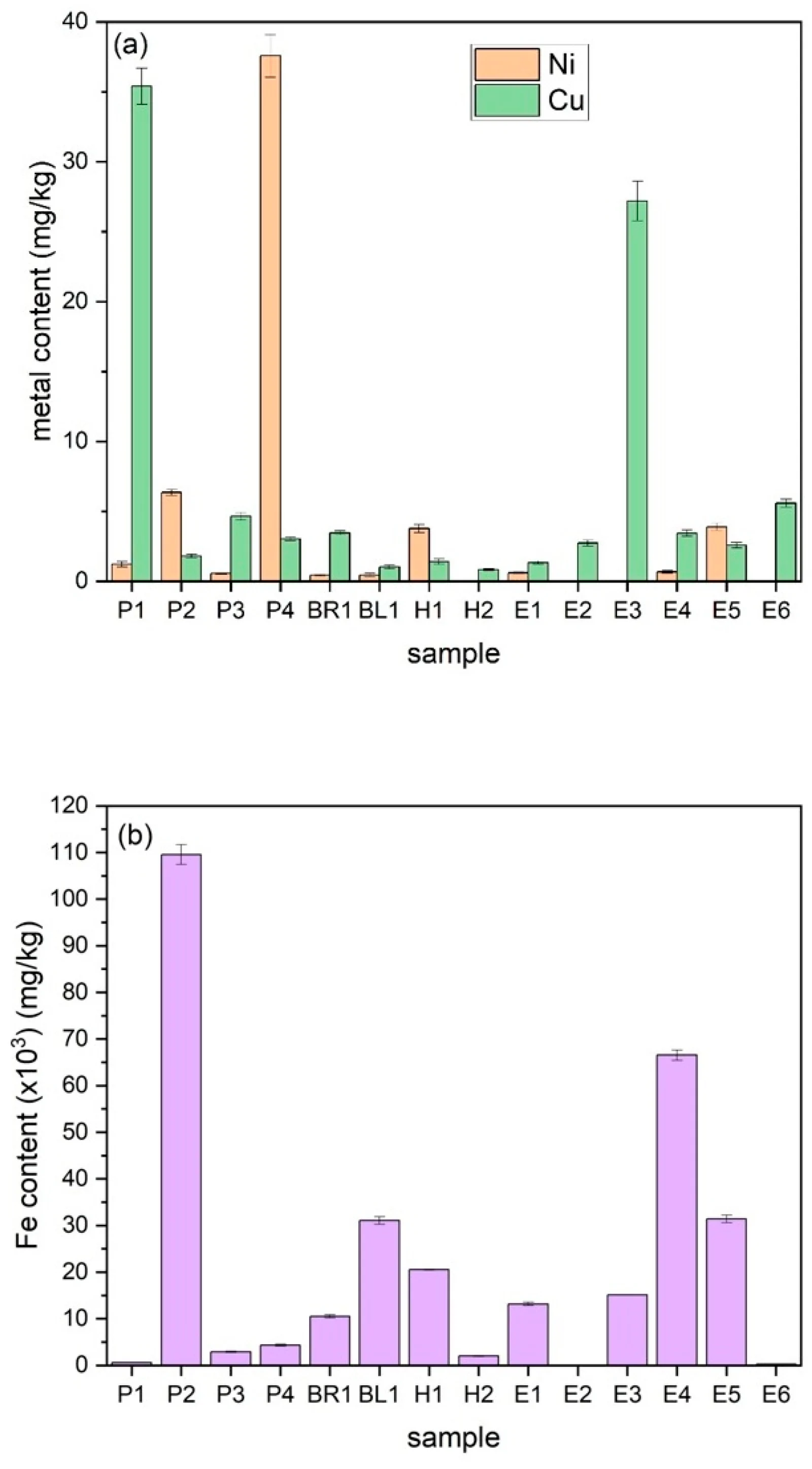
Concentrations of the analyzed metals in the tested cosmetic samples (mg/kg): (**a**) nickel and copper; (**b**) iron. Data are presented as the mean ± standard deviation. P1–P4, face powders; BR1, bronzer; BL1, blush; H1–H2, highlighters; E1–E6, eyeshadows.

The concentrations of the analyzed metals in the tested cosmetic samples are presented in Figure 1. For clarity, the concentrations of nickel and copper are shown together (Figure 1a), whereas the concentrations of iron are presented separately (Figure 1b) because of the substantial differences in their concentration ranges.

The presence of nickel (Table 3) was confirmed in all analyzed cosmetic samples except for four, in which its concentration was below the limit of detection (highlighter H2 and eyeshadows E2, E3, and E6). In face powders, nickel concentrations ranged from 0.56 to 37.57 mg/kg. Among the contouring products, nickel was below the limit of detection in one sample, whereas in the remaining products its concentration ranged from 0.45 to 3.76 mg/kg. Similarly, nickel was below the limit of detection in half of the eyeshadow samples, while in the remaining samples its concentration ranged from 0.60 to 3.89 mg/kg. Among all the cosmetics analyzed, the highest nickel concentration was found in face powder P4 (37.57 ± 0.53 mg/kg); according to Tukey’s post hoc test, it differed statistically significantly from all other tested cosmetics (*p* < 0.001). Face powder P2 exhibited the second-highest nickel content, which was statistically distinct from both P4 and the remaining lower-concentration products (*p* < 0.001). Furthermore, highlighter H1 and eyeshadow E5 did not differ significantly from each other (*p* = 1.000), forming a shared homogenous group that differed significantly from all other tested cosmetics (*p* < 0.001). No statistically significant differences were observed among the samples characterized by low nickel levels—such as bronzer BR1, blush BL1, highlighter H2, and eyeshadows E1–E4 and E6—indicating a uniform baseline profile for the majority of the analyzed items. The categorical analysis for nickel revealed that face powders possessed the highest group mean. Tukey’s test confirmed that the nickel content in powders was significantly higher than in both contouring products (*p* = 0.015) and eyeshadows (*p* = 0.006). In contrast, the difference between contouring products and eyeshadows was not statistically significant (*p* = 0.996).

The presence of copper (Table 3) was confirmed in all analyzed cosmetic samples. In face powders, copper concentrations ranged from 1.80 to 35.39 mg/kg. In the contouring products, copper concentrations ranged from 0.83 to 3.46 mg/kg. Similarly, in eyeshadows, copper concentrations ranged from 1.33 to 27.19 mg/kg. Among all the cosmetics analyzed, the highest copper concentration was found in face powder P1 (35.39 ± 0.30 mg/kg), whereas the lowest concentration was recorded in highlighter H2 (0.83 ± 0.07 mg/kg). According to Tukey’s post hoc test, face powder P1 exhibited a unique contamination profile, differing significantly from all other evaluated samples (*p* < 0.001). Similarly, eyeshadow E3, which contained the second-highest copper concentration, formed an independent statistical group with significant differences compared to all remaining samples (*p* < 0.001). A strong statistical similarity was observed within intermediate-concentration clusters. Face powder P4 (3.02 mg/kg) showed no significant differences when compared to bronzer BR1 (*p* = 0.999), eyeshadow E2 (*p* > 0.999), eyeshadow E4 (*p* > 0.999), and eyeshadow E5 (*p* = 0.999). Regarding the samples with the lowest copper content, a complex pattern of overlapping homogeneous groups was revealed. Face powder P2 displayed strong statistical commonality with blush BL1 (*p* = 0.885), highlighter H1 (*p* > 0.999), highlighter H2 (*p* = 0.637), and eyeshadow E1 (*p* = 0.998). However, categorical analysis of copper concentrations across the evaluated cosmetic groups revealed no statistically significant differences. Tukey’s post hoc test confirmed that the copper content in face powders did not differ significantly from either contouring products (*p* = 0.061) or eyeshadows (*p* = 0.520). Furthermore, no statistically meaningful variation was observed between contouring products and eyeshadows (*p* = 0.314).

The presence of iron (Table 3) was confirmed in all analyzed cosmetic samples. In face powders, iron concentrations ranged from 0.61 × 10^3^ to 109.56 × 10^3^ mg/kg. In the contouring products, iron concentrations ranged from 1.99 × 10^3^ to 31.09 × 10^3^ mg/kg. Similarly, in eyeshadows, iron concentrations ranged from 0.03 × 10^3^ to 66.55 × 10^3^ mg/kg. Among all the cosmetics analyzed, the highest iron concentration was found in face powder P2 (109.56 × 10^3^ ± 2.14 × 10^3^ mg/kg), whereas the lowest concentration was recorded in eyeshadow E2 (0.03 × 10^3^ ± 0.01 × 10^3^ mg/kg). Tukey’s post hoc test revealed highly significant variations in iron content across the evaluated samples. Face powder P2, containing the highest absolute iron concentration, differed significantly (*p* < 0.001) from all other analyzed products, establishing a distinct statistical group. Conversely, eyeshadow E2 (the sample with the lowest iron content) exhibited strong homogeneity with face powder P1 (*p* = 0.998) and eyeshadow E6 (*p* > 0.999), while remaining significantly different from the rest of the sample matrix. A complex pattern of intermediate clusters was observed. Blush BL1 and eyeshadow E5 showed near-perfect statistical identity (*p* > 0.999). Face powder P3 did not differ significantly from powder P4 (*p* = 0.457) or highlighter H2 (*p* = 0.929); however, a significant difference was confirmed between P4 and H2 (*p* = 0.018). Eyeshadow E1 displayed a lack of statistically significant variance when compared to E3 (*p* = 0.076). In contrast, samples BR1, H1, and eyeshadow E4 exhibited highly unique concentration profiles, demonstrating significant differences (*p* < 0.005) against all other evaluated cosmetic products. When the analyzed samples were categorized into three distinct functional groups: face powders, contouring products, and eyeshadows, Tukey’s post hoc test revealed no significant differences in iron content among the evaluated cosmetic groups. The variance in iron levels between face powders and contouring products was found to be statistically non-significant (*p* = 0.541). Similarly, no statistically significant differences were observed when comparing eyeshadows to either face powders (*p* = 0.751) or contouring products (*p* = 0.898).

### 3.3. Correlation Analysis Between the Analyzed Metals

Pearson correlation coefficients (r) were calculated globally based on the mean concentration values of the 14 independent commercial cosmetic products to evaluate potential linear relationships among nickel, copper, and iron. The statistical evaluation revealed that none of the evaluated pairs of metals exhibited a statistically significant correlation (*p* ≥ 0.05). Globally, extremely weak and statistically non-significant correlations were found between Ni and Cu (r = −0.135), Ni and Fe (r = −0.024), as well as between Cu and Fe (r = −0.232). These results demonstrate the absence of a strong global linear relationship among the concentrations of the investigated metals across the analyzed formulations.

### 3.4. Principal Component Analysis (PCA)

**Figure 2:**
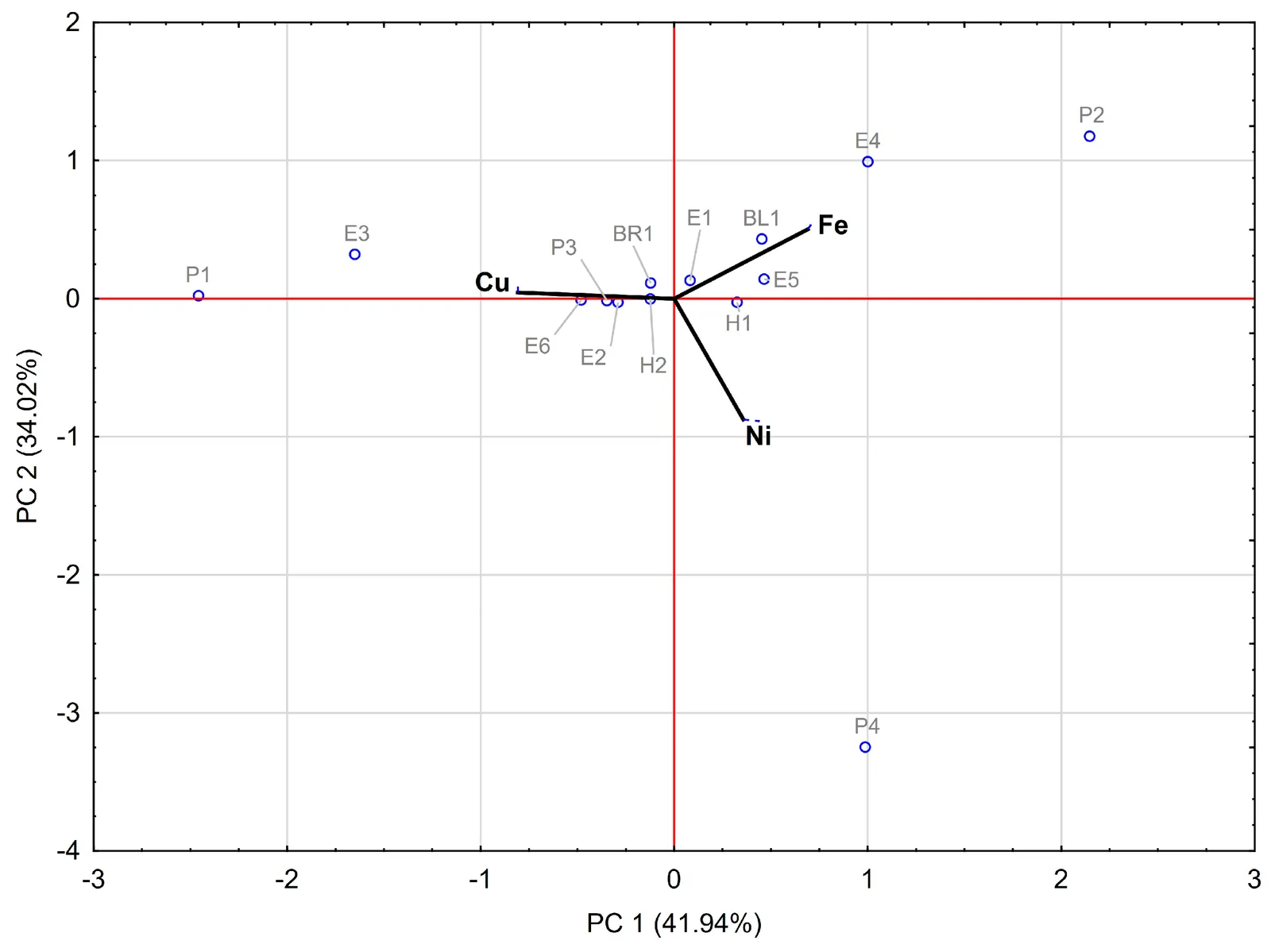
Principal Component Analysis (PCA) biplot demonstrating the simultaneous projection of chemical loading vectors (Ni, Cu, Fe) and factor scores for the evaluated cosmetic samples (P1–P4, face powders; BR1, bronzer; BL1, blush; H1–H2, highlighters; E1–E6, eyeshadows). The horizontal (PC1) and vertical (PC2) axes account for 41.94% and 34.02% of the total variance, respectively.

The multivariate relationship between the analyzed cosmetic categories and their metal profiles was further evaluated using Principal Component Analysis. The resulting biplot (Figure 2) illustrates the simultaneous projection of chemical loadings, including nickel, copper, and iron vectors, and factor scores for the fourteen commercial samples on the two-dimensional factor plane. The first two principal components explained 41.94% of the total dataset variance for PC1 and 34.02% for PC2, respectively, yielding a high cumulative variance of 75.96%, which ensures a highly reliable representation of the multi-element composition. The spatial distribution of the cosmetic items on the factor plane revealed a distinct chemical stratification directly governed by specific metallic anomalies. A prominent cluster of standard formulations comprising eyeshadows E1, E2, and E6, highlighter H2, face powder P3, and bronzer BR1 gathered tightly near the plot origin, demonstrating a highly homogeneous, low-level baseline metal profile. In contrast, extreme outlier products exhibited strong vector-driven migration across different quadrants of the biplot. Face powder P2 and eyeshadow E4 shifted heavily into the upper-right quadrant, demonstrating a powerful correlation with the iron vector, which reflects their heavily pigmented oxide matrices. Conversely, face powder P4 migrated vertically downward along the negative PC2 axis, mirroring the exact trajectory of the nickel vector and highlighting its critical contamination anomaly, while eyeshadow E5 and highlighter H1 also aligned with this directional pull to a lesser extent. Furthermore, face powder P1 and eyeshadow E3 were strongly pulled into the upper-left quadrant along the negative PC1 axis, driven exclusively by the copper loading vector.

### 3.5. Hierarchical Cluster Analysis (HCA)

**Figure 3:**
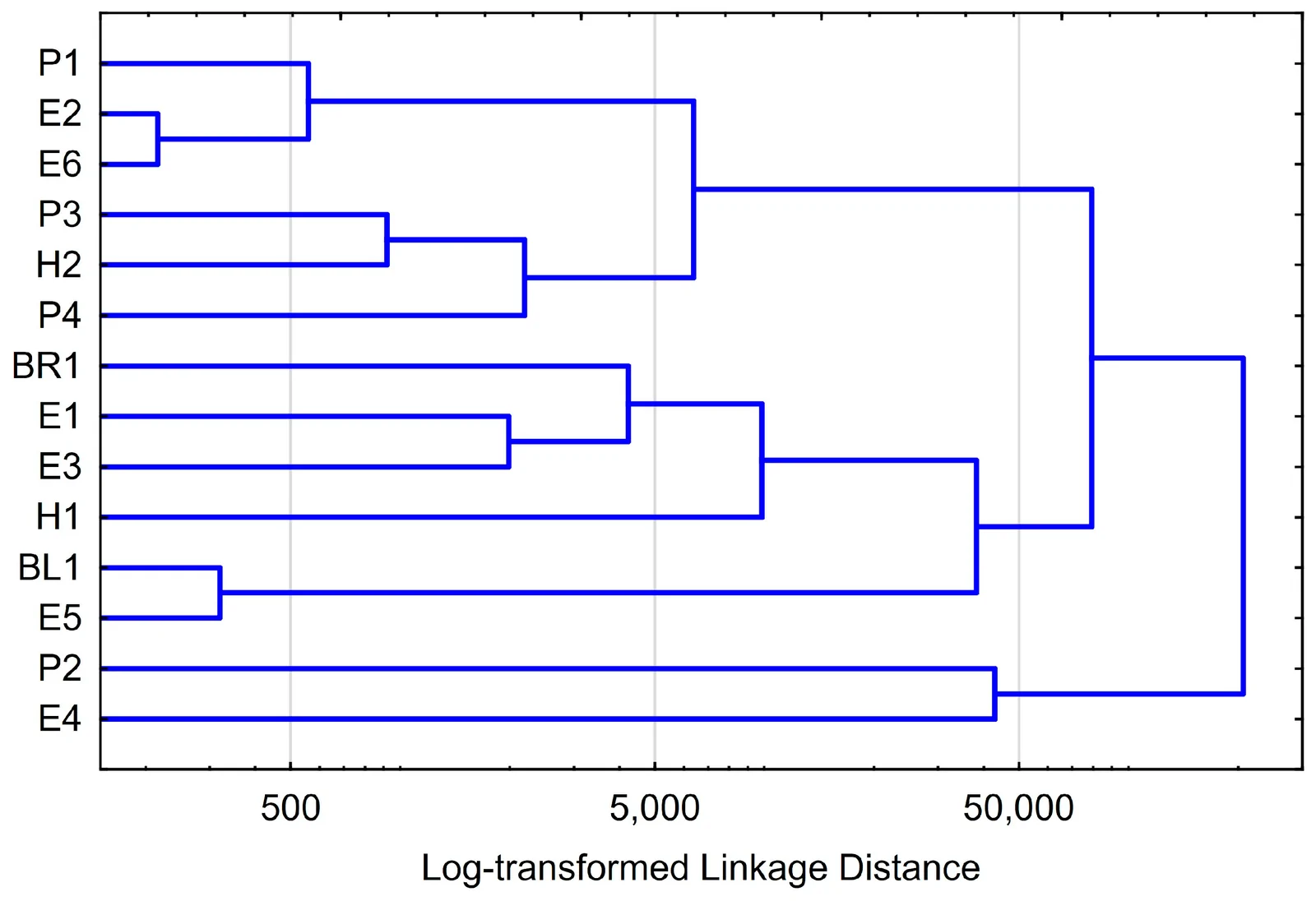
Hierarchical cluster analysis (HCA) dendrogram of the fourteen evaluated cosmetic samples (P1–P4, face powders; BR1, bronzer; BL1, blush; H1–H2, highlighters; E1–E6, eyeshadows) generated using Ward’s clustering method and Euclidean distance with a log-transformed linkage distance scale.

To complement the multivariate relationships established by PCA, hierarchical cluster analysis was performed on the cosmetic dataset. Agglomerative clustering was executed using Ward’s minimum variance method as the linkage criterion, with Euclidean distance serving as the proximity measure to minimize within-cluster variance. Due to the substantial order-of-magnitude variations among the metal concentrations, a log-transformed linkage distance was utilized on the primary clustering axis to enhance topological resolution and ensure macro- and micro-compositional profiles were evaluated with equal statistical sensitivity. The resulting dendrogram (Figure 3) illustrated a highly structured hierarchical topology that perfectly corroborated the spatial distribution patterns identified in the PCA model. At the highest level of linkage distance separation, the evaluated cosmetics were resolved into two primary, distinct branches. The lowest sub-branch distinctly isolated face powder P2 and eyeshadow E4, establishing a separate cluster that reflects their unique compositional status driven by massive iron formulation matrices, primarily associated with intentionally added iron-based pigments rather than accidental contamination. The upper main branch was further subdivided into distinct categorical nodes based on intermediate and baseline metal patterns. Within this structure, face powder P4, face powder P3, and highlighter H2 formed a tightly linked sub-cluster, while eyeshadows E2 and E6 paired directly with face powder P1, demonstrating a strong proximity pattern. Another distinct cluster successfully aggregated blush BL1 and eyeshadow E5 at a remarkably low linkage distance, showing near-perfect composition homogeneity, which then linked progressively with highlighter H1, eyeshadow E3, eyeshadow E1, and bronzer BR1. This extensive, dense agglomeration of the majority of eyeshadows, highlighters, and contouring products at lower linkage distance thresholds confirms the presence of a uniform, non-differentiated baseline profile across standard commercial formulations. Importantly, these HCA clustering patterns describe similarities in overall elemental profiles determined by the intentional formulation composition (such as pigment loading or specific active components like copper gluconate) rather than differences in product purity or impurity/contamination levels.

### 3.6. Quantitative Health Risk Assessment Results

The calculated CDDE, HQ, and HI values for the evaluated cosmetic products are summarized in Table 4. Nickel concentrations were below the limit of detection (LOD) in four cosmetic samples; therefore, these samples were excluded from the subsequent health risk assessment for this element. Among the remaining samples, the highest chronic daily exposure (CDDE) of Ni was recorded for face powder P4, whereas the lowest was recorded for eyeshadow E1. For Cu, the highest CDDE was recorded for face powder P1, while the lowest was observed for eyeshadow E1. For Fe, the highest CDDE was recorded for face powder P2, whereas the lowest was recorded for eyeshadow E2. The HQ values for all analyzed elements in all cosmetic samples were below the safety threshold (HQ < 1). Furthermore, the cumulative Hazard Index (HI) remained below the safety threshold (HI < 1) for all tested cosmetics, indicating that combined exposure to Ni, Cu, and Fe is not expected to pose a significant non-carcinogenic health risk under the assumed exposure conditions.

The lifetime cancer risk (LCR) determined for Ni in the cosmetic products where it was detected varied between 5.63 × 10^−12^ (eyeshadow E1) and 8.28 × 10^−9^ (powder P4), as detailed in Table 5. These results indicate that the carcinogenicity risk associated with the tested cosmetics remains within tolerable thresholds.

## 4. Discussion

The presence of heavy metals in cosmetic products is associated with potential risks to human health; therefore, this topic has attracted considerable attention from researchers worldwide. Studies on their occurrence in cosmetics are essential to ensure product safety [41].

The research topic addressed in the present study introduces a significant element of novelty to both the national and European literature in the field of cosmetic toxicology. Although the scientific literature extensively describes the levels of contamination and release profiles of heavy metals from liquid, semi-liquid, and emulsion-based cosmetic products [20,21,22,23,24], as well as selected color cosmetics such as eyeshadows [23,25,26,27,28], integrated studies covering different categories of loose cosmetic forms (face powders, contouring products, and eyeshadows) remain relatively scarce, particularly in relation to the Polish retail market [24,25]. The specific characteristics of loose cosmetic products, which are defined by a high degree of solid-phase dispersion, result in a distinctly different raw material and technological risk profile compared to formulations with a continuous liquid phase [13,24]. The present study offers a preliminary comparative assessment of metal profiles in loose cosmetic products, focusing on their intended use and application characteristics. Notably, investigating the potential presence of metals like Ni, Cu, and Fe offers preliminary insights that can support the broader evaluation of quality control standards for cosmetic products. The obtained results indicate that, despite the strict regulatory framework of Regulation (EC) No. 1223/2009 [5], technological limitations in raw material purity persist in real market conditions, making the monitoring of unintended contamination in loose cosmetic products essential for ensuring consumer safety [4,24].

### 4.1. Nickel

Nickel functions strictly as an unintentional, technologically unavoidable raw-material impurity across all functional categories due to its absolute regulatory prohibition under the European cosmetics framework [5]. In the present study, nickel was detected in the majority of face powders, with concentrations ranging from 0.56 to 37.57 mg/kg, presenting a statistically higher group mean compared to contouring products and eyeshadows (*p* < 0.05).

Cross-referencing our data with the global literature reveals high variability in powder matrices. Our results for face powders are notably higher than the low concentration ranges reported by Ahmed et al. [22], UkoNaku et al. [42], and Ullah et al. [43], while remaining well below the maximum contamination peaks reported by Orisakwe and Otaraku (2.50–19.70 mg/kg) [44] and Omenka and Adeyi (0.13–107.00 mg/kg) [45]. The elevated nickel content (37.57 mg/kg) observed in powder P4 may be explained by two most probable sources of contamination. First, nickel may originate from mineral-based cosmetic raw materials, particularly pigments and fillers. Iron oxides, titanium dioxide, and other naturally derived mineral ingredients are known to contain trace amounts of nickel as an impurity inherited from the parent ores or introduced during pigment production. Consequently, the nickel concentration in the final cosmetic product may vary depending on the source and purity of these raw materials [13]. Second, the presence of rice powder, listed among the ingredients of product P4, may also contribute to the elevated nickel concentration. Rice may accumulate trace elements, including nickel, from soil and irrigation water, and the elemental composition of rice-derived materials depends strongly on soil geochemistry, environmental conditions, water quality, and agricultural practices. As a result, rice powder may contain variable concentrations of nickel depending on its geographical origin and cultivation conditions [46,47,48]. Without detailed information on the origin of the raw materials and the manufacturing process, the primary source of nickel cannot be conclusively identified.

For face contouring products, bronzer BR1 and blush BL1 exhibited low levels (0.45 mg/kg) compared to the higher blush ranges found by Kicińska and Kowalczyk (3.79–95.64 mg/kg) [25] and Adam-Dima et al. (7.34–19.10 mg/kg) [21]. In highlighters, our results were lower than those of Ahmed et al. (4.45–8.22 mg/kg) [22]. For eyeshadows, our heterogeneous findings (LOD to 3.89 mg/kg) fall within the intermediate boundaries established by Alvarez-Gonzalez et al. (1.53–4.73 mg/kg) [23], Kicińska and Kowalczyk (3.48–7.97 mg/kg) [25], Adam-Dima et al. (1.86–10.1 mg/kg) [21], and Voica et al. (1.72–30.62 mg/kg) [26], while remaining far below the broad limits of Sainio et al. (0.05–49.7 mg/kg) [49], Hepp et al. (0.91–42.00 mg/kg, with an extreme outlier at 1600.00 mg/kg) [27], and significantly above the trace levels of Mayildurai et al. (0.01–0.04 mg/kg) [50].

Nickel exhibits the ability to bind to keratin and accumulate in the stratum corneum, which is directly associated with the induction of allergic reactions. Moreover, this element is classified as a carcinogenic substance and has been shown to exert neurotoxic effects [24]. The toxicological significance of these findings shifts from systemic risk to localized cutaneous reactivity. Because the calculated lifetime cancer risk (LCR) for nickel in all positive samples remained multiple orders of magnitude below the conservative regulatory threshold of 10^−6^ (Table 5), systemic carcinogenicity via the dermal route is toxicologically negligible [22,23]. Instead, the primary health hazard identified in this screening is the potential for inducing or triggering allergic contact dermatitis, as nickel is a potent contact allergen and a well-characterized skin sensitizer [49].

From a pathophysiological perspective, embedded nickel oxides must undergo oxidation and subsequent dissolution upon contact with human sweat, releasing free Ni^2+^ ions. These divalent cations act as haptenic agents, penetrating the lipophilic stratum corneum barrier to bind binding proteins in the viable epidermis. This complex activates the Toll-like receptor 4 pathway on antigen-presenting cells, initiating a Type IV delayed-type hypersensitivity reaction [51]. The maximum concentration discovered in face powder P4 (37.57 ± 0.53 mg/kg) is highly critical in this context.

Clinical and dose–response evaluations under realistic exposure conditions indicate that while the vast majority of sensitized individuals do not react to nickel levels below 10 mg/kg, a maximum safety threshold of 5 mg/kg has been recommended for consumer products to prevent elicitation, with 1 mg/kg established as the ultimate target for comprehensive protection [52]. Furthermore, loose cosmetic formulations are applied via mechanical buffing with brushes, which can induce micro-abrasions in the stratum corneum, compromising the skin barrier and significantly accelerating ion penetration [53]. Consequently, the high nickel concentration in sample P4 represents a genuine dermatological risk for the sensitized cohort of the population, confirming that monitoring raw material purity remains an indispensable requirement for local tolerance management.

### 4.2. Copper

In the present study, copper concentrations across the evaluated matrix revealed a distinct operational duality. In the majority of products, copper functioned strictly as an accidental, technically unavoidable background trace contaminant embedded within mineral substrates (ranging from 0.83 to 5.59 mg/kg). This baseline contamination profile is higher than the trace levels reported by Ahmed et al. (0.15–0.65 mg/kg) [22] and Ullah et al. (1.09–1.51 mg/kg) [43], but remains vastly inferior to the exceptional contamination peak of 459.54 mg/kg reported by Łodyga-Chruścińska et al. [54]. Conversely, copper represents an intentionally incorporated skincare active ingredient exclusively in face powder P2, which declares the presence of Copper Gluconate in its official INCI composition.

From a dermatological and pathophysiological perspective, the chemical speciation of copper determines its local safety profile. Metallic copper possesses a remarkably low allergenic and sensitization potential compared to nickel. However, when accidental copper contaminants undergo ambient oxidation, they can release free divalent copper ions (Cu^2+^). Upon contact with human sweat, these free ions can act as local skin irritants rather than true sensitizers, especially if applied to a compromised epidermal barrier [55]. The biochemical mechanism of this toxicity is primarily driven by the ability of free Cu^2+^ ions to participate in Fenton-like chemical reactions. This process generates highly reactive hydroxyl radicals, inducing localized oxidative stress, lipid peroxidation of cell membranes, and subsequent acute cutaneous inflammation characterized by pruritus and erythema [55].

In sharp contrast, the intentional use of Copper Gluconate in sample P2 represents a safe, chemically stabilized chelate structure. Cosmetic safety assessments of Copper Gluconate confirm that it is non-irritating and non-sensitizing at commercial concentrations, functioning as a structural support agent that accelerates skin regeneration and collagen synthesis [56].

### 4.3. Iron

In evaluating the toxicological and technological profile of iron, it is critical to emphasize that this element cannot be classified strictly as a heavy metal contaminant, but must be assessed based on its primary function as a major cosmetic ingredient.

In this study, iron concentrations in 3 analyzed powder samples ranged from 0.61 × 10^3^ to 4.33 × 10^3^ mg/kg, whereas one product (P2) exhibited a markedly elevated concentration of 109.56 × 10^3^ mg/kg. The evaluation of the declared INCI frameworks demonstrates that iron functions predominantly as an intentionally incorporated structural component and mineral colorant rather than a contaminant in twelve out of the fourteen evaluated loose cosmetics. Its massive elemental loads are directly introduced via the mineral iron oxide pigments declared on the product labels. Conversely, in the pigment-free face powder P4 and eyeshadow E6, iron oxides are entirely absent from the official INCI declarations, meaning that these low detected iron concentrations represent genuine, non-intentional background processing impurities. The value determined for sample P2 exceeded both the concentrations obtained in the present study for the remaining samples and those reported in the literature. Ahmed et al., analyzing 10 face powders, reported iron concentrations ranging from 10.15 to 98.22 mg/kg [22]. Higher concentrations than those reported by Ahmed et al. were found by Ullah et al., who reported iron levels ranging from 0.65 × 10^3^ to 1.07 × 10^3^ mg/kg [43]. In comparison, Łodyga-Chruścińska et al. determined iron concentrations ranging from 4.18 × 10^3^ to 12.17 × 10^3^ mg/kg in 6 analyzed samples [54].

The iron concentrations determined in the face contouring products analyzed in the present study were 10.48 × 10^3^ mg/kg for bronzer, 31.09 × 10^3^ mg/kg for blush, and 1.99 × 10^3^ and 20.52 × 10^3^ mg/kg for the two highlighter samples, respectively. The value obtained for blush falls within the wide range reported by Kicińska and Kowalczyk (0.54 × 10^3^–16.56 × 10^3^ mg/kg for 4 blush samples) [25]. In contrast, the iron content profile of highlighters was several times higher compared to the low and narrow range reported by Ahmed et al., who determined iron levels of only 1.13–6.21 mg/kg in 10 analyzed highlighters [22].

In the eyeshadow group, a wide range of iron concentrations was observed, ranging from 33.30 to 66.55 × 10^3^ mg/kg. The high levels of iron content in this type of cosmetic are supported by the literature. A comparable concentration profile was reported by Alvarez-Gonzalez et al., who determined iron levels in 12 eyeshadow samples in the range of 1 0.39 × 10^3^–97.67 × 10^3^ mg/kg [23]. A somewhat narrower but still elevated concentration range was reported by Kicińska and Kowalczyk, who found iron levels of 6052.00–24,882.00 mg/kg in 3 analyzed eyeshadows [25]. Particularly high values were reported by Meena et al., who analyzed 6 eyeshadow samples and found iron concentrations ranging from 5.44 × 10^3^ to as high as 475 × 10^3^ mg/kg [28].

From a toxicological standpoint, the high concentrations of iron oxides in pigmented formulations do not automatically imply a significant systemic health hazard via the dermal route. These mineral colorants are generally recognized as chemically inert substances under normal conditions of use [57]. This status is further supported by the calculated dermal Hazard Quotients, which remained multiple orders of magnitude below the safety threshold across all tested samples (maximum HQ = 8.84 × 10^−5^). Consequently, under the specific exposure assumptions utilized in this dermal screening model, the chronic systemic risk associated with iron in these formulations appears to be negligible for the consumer.

### 4.4. Toxicological and Technological Implications of Categorical and Multivariate Trends

The extensive statistical screening performed in this study provides crucial insights into the origin and nature of metal occurrences in loose cosmetic products. The integration of univariate (ANOVA and Tukey’s HSD test) and multivariate (HCA and PCA) analyses successfully identified individual high-risk products, showing that specific face powders (P1, P2, P4) and eyeshadows (E3, E4) act as distinct concentration peaks for individual elements. Categorical statistical comparison revealed that face powders present a significantly higher risk of nickel contamination compared to eyeshadows and contouring products, whereas copper and iron levels overlap across categories due to high intra-group variance. These findings indicate that while individual cosmetic formulations exhibit highly specific and significantly diverse iron concentrations, the overall functional category of the cosmetic (powder, contouring, or eyeshadow) does not act as a determining factor for the total iron burden. This lack of significance suggests a wide and overlapping distribution of iron-based pigments across all three product types, rather than a category-specific accumulation pattern.

From a toxicological and technological perspective, the universal lack of statistically significant Pearson correlations suggests that the presence of nickel, copper, and iron in the studied cosmetic matrices may not originate from a single, shared source of raw mineral contamination or accidental bulk pollution during manufacturing. Instead, the accumulation of these elements appears to be highly variable and potentially dependent on specific product-by-product formulations. The results indicate that the concentration of major mineral pigments (represented by iron oxide) does not serve as a reliable predictor for trace impurities like nickel or copper, which points toward potentially distinct entry pathways for these metals.

This hypothesis regarding independent entry mechanisms is further supported by the multivariate statistical treatments. The clear spatial separation on the PCA biplot (Figure 2) indicates that the metal occurrences in the studied cosmetics do not stem from a uniform source of raw materials, but are instead likely dictated by independent factors. For instance, the extreme concentrations of iron observed in face powder P2 (109.56 × 10^3^ mg/kg) and eyeshadow E4 (66.55 × 10^3^ mg/kg), which isolated them as distinct outliers in both the PCA biplot (Figure 2) and HCA dendrogram (Figure 3), are directly related to their functional roles. Iron oxides (Fe_2_O_3_, Fe_3_O_4_, and FeOOH) are the primary, legally approved inorganic pigments used to achieve red, yellow, black, and brown shades in decorative cosmetics [2,19]. Consequently, their massive presence represents a structural, intentional formulation component rather than an accidental impurity [4].

In contrast, nickel and copper (with the exception of the copper-containing active ingredient declared in P2) enter cosmetic matrices strictly as unintended trace impurities. Natural mineral bases, such as talc (magnesium silicate), mica (potassium aluminum silicate), and kaolin clay, are extracted from the Earth’s crust where they co-occur with natural geochemical deposits of transition metals [2,13]. The global absence of significant correlations (*p* ≥ 0.05) between iron and the other trace impurities (Ni, Cu) suggests that the primary iron oxide colorants are likely not the major vehicle for toxic nickel or copper entry. Instead, the contamination pathways may vary independently, potentially originating from the varying quality, geological source, or purification efficiency of the specific talc or mica batches used as the powder substrate [13,24]. This is further illustrated by face powder P4, which migrated toward a completely different spatial vector due to its unique nickel spike (37.57 mg/kg), completely independent of its iron or copper content. Such high localized nickel contamination poses a notable dermatological risk, as nickel is a well-known potent contact allergen capable of triggering allergic contact dermatitis even at low trace thresholds upon regular skin contact [13,21].

Importantly, the dense clustering of the majority of bronzers, blushes, highlighters, and eyeshadows at very short linkage distances in the HCA tree diagram (Figure 3) indicates a high degree of compositional similarity among the standard formulations investigated in this study. For the majority of these analyzed products, the baseline elemental background remains uniformly low. This suggests that while the selected sample set displays consistent baseline profiles, potential exposure variations appear to be highly sample-specific and restricted to particular formulations (such as P1, P2, P4, and E3) where distinct raw material matrices or mineral properties play a dominant role [24,25]. Therefore, safety assessments cannot rely on category-wide generalizations; instead, regular, multi-element independent monitoring of specific batches remains an indispensable requirement for regulatory compliance, minimization of human exposure, and long-term consumer health protection [4,24].

### 4.5. Health Risk Characterization and Perspectives

All cosmetic products analyzed in this study were purchased on the Polish retail market, which means that they are subject to the strict requirements of Regulation (EC) No 1223/2009 of the European Parliament and of the Council on cosmetic products. According to Annex II of this regulation, the intentional use of nickel and its compounds in cosmetic formulations is strictly prohibited. However, the regulation permits the unintentional presence of trace amounts of prohibited substances, provided that such contamination is technologically unavoidable (e.g., originating from impurities in raw materials or the synthesis process of pigments) and safe for human health [5]. The detected levels of Ni across all samples, as well as Cu (with the exception of powder P2), indicate that these metals occur as non-intentional, technically unavoidable impurities derived from mineral raw materials. In contrast, the presence of Fe across the majority of samples, as well as Cu in powder P2, is consistent with intentionally added ingredients specified in their INCI compositions, such as iron oxide pigments (e.g., CI 77491, CI 77492, CI 77499), ferric ferrocyanide (CI 77510 in sample E3), and copper gluconate. Crucially, for samples devoid of iron-based pigments or copper compounds in their declared INCI lists (such as face powder P4 and eyeshadow E6), these metals represent impurities. Therefore, the origin of Fe and Cu must be interpreted on a product-specific basis according to the declared formulation matrix, whereas Ni represents an impurity across all analyzed items. In this regulatory context, a quantitative risk assessment utilizing HQ, HI, and LCR does not replace the comprehensive cosmetic product safety assessment legally mandated under Regulation (EC) No 1223/2009 [5], which systematically evaluates product composition, ingredient function, local tolerance, stability, microbiological quality, and labeling. Instead, these mathematical indices serve as a valuable quantitative screening-level tool to objectively verify systemic consumer safety regarding technically unavoidable impurities under real-world market conditions.

However, a primary methodological boundary of the quantitative screening executed in this work is its exclusive reliance on the dermal absorption pathway for nickel, copper, and iron. Expanding the exposure algorithms to include quantitative involuntary inhalation and accidental ingestion for loose powder matrices is restricted by significant data gaps in standardized toxicological repositories. Although the application of fine, uncompacted powders using cosmetic brushes inevitably generates a temporary particulate aerosol cloud within the consumer’s breathing zone, standard evaluation frameworks do not provide validated constants to support multi-route mathematical modeling in this product category. Specifically, the United States Environmental Protection Agency Integrated Risk Information System (USEPA IRIS) database [58] does not establish non-carcinogenic consumer inhalation reference concentrations or reference doses for copper, iron, and soluble nickel salts, rendering multi-route Hazard Quotient estimations mathematically impossible. The inhalation carcinogenicity factors available in official repositories are restricted exclusively to industrial occupational matrices like nickel refinery dust, which do not reflect the refined mineral or organic ingredients utilized in commercial cosmetics. Furthermore, the European Commission Scientific Committee on Consumer Safety (SCCS) guidelines [32] parameterize accidental product ingestion models exclusively for oral hygiene products and lip cosmetics, completely omitting ingestion constants for facial makeup applied outside the perioral zone. Consequently, due to the absence of standardized aerodynamic mass emission rates, particle size distribution profiles under consumer use, and systemic ingestion variables, the dermal pathway remains the only viable and reproducible route for establishing screening-level benchmarks. To mitigate these exposure uncertainties, this dermal health risk assessment was intentionally designed under a strict worst-case scenario principle, assuming direct product application to bare skin without the mitigating barrier effects of a wet foundation base. Because the calculated cumulative Hazard Index for all evaluated elements remained multiple orders of magnitude below the critical benchmark (HI < 1) under these maximized assumptions, the model successfully functions as a protective upper-bound threshold specifically for dermal exposure. Consequently, while the current dermal screening provides a valuable initial baseline for safety characterization, these data highlight the importance of future aerodynamic and multi-route exposure studies tailored specifically for loose cosmetic formulations. Such advancements will be vital to bridge existing methodology gaps and fully refine comprehensive, multi-route risk assessment models for consumer products.

#### 4.5.1. Non-Carcinogenic Risk Assessment

For nickel, the highest CDDE and HQ values were recorded for powder P4 (2.12 × 10^−8^ mg·kg^−1^·day^−1^ and 3.93 × 10^−6^, respectively). Although the systemic risk is negligible, the presence of nickel in cosmetic products requires monitoring due to its allergenic properties. Nickel is the most common cause of allergic contact dermatitis. Even trace concentrations may induce local reactions in hypersensitive individuals [21]. In contrast, the highest CDDE for copper was observed in powder P1 (2.00 × 10^−8^ mg·kg^−1^·day^−1^); however, the HQ value of 5.00 × 10^−7^ confirms the systemic safety of the analyzed cosmetics. Iron exhibited the highest CDDE values among all analyzed metals, reaching a maximum for powder P2 (6.19 × 10^−5^ mg·kg^−1^·day^−1^ and HQ = 8.84 × 10^−5^). High iron concentrations are typical for colored cosmetics (such as face powders, contouring products, and eyeshadows), where iron oxides are commonly used as mineral pigments providing yellow, red, and black coloration. Nevertheless, the low HQ values indicate that dermal absorption of iron does not pose a health risk to the human body.

A key safety criterion in US EPA models is the Hazard Quotient (HQ) and Hazard Index (HI), where values below unity (HQ < 1, HI < 1) indicate no significant toxic risk [23,34]. In this study, the highest HI value was 8.91 × 10^−5^ (for powder P2), which is several orders of magnitude lower than the safety threshold. Therefore, the obtained results indicate that exposure to Ni, Cu, and Fe through the use of the analyzed cosmetic products does not pose a non-carcinogenic health risk to consumers.

#### 4.5.2. Carcinogenic Risk Assessment

The lifetime cancer risk (LCR) for nickel (in cosmetics where its presence was confirmed) ranged from 5.63 × 10^−12^ (eyeshadow E1) to 8.28 × 10^−9^ (powder P4). These values are below 10^−6^ and are therefore considered safe and acceptable for daily consumer exposure [25,40]. Higher LCR (as well as CDDE) values for face powders compared to eyeshadows result from differences in application patterns; face powders are applied over a larger surface area of the face, which in the mathematical model leads to a higher estimated daily dose. It should be noted that although nickel is classified as a carcinogenic agent primarily via inhalation exposure, the estimation of LCR for dermal exposure provides a conservative and precautionary approach to consumer safety assessment [22,23].

## 5. Conclusions

The presented study enabled a comprehensive screening-level assessment of selected metals (Ni, Cu, Fe) in loose color cosmetics, including face powders, contouring products, and eyeshadows available on the Polish retail market, as well as an evaluation of the health risk associated with their use. The applied microwave-assisted digestion and MP-AES procedure was validated and proved suitable for reliable determination of these elements in the analyzed cosmetic matrices. The INCI list information of the analyzed samples allowed a more reliable interpretation of the detected Fe and Cu. Based on the declared ingredients, iron-containing pigments or other iron-based ingredients were present in the majority of the analyzed products, with the exception of powder sample P4 and eyeshadow sample E6, whereas a copper-containing component was declared only for powder sample P2. Consequently, in products containing declared iron- or copper-based ingredients, these metals may be associated with intentionally added components, whereas in products without such declarations, their presence is more likely attributable to contaminants. Nevertheless, the contribution of impurities cannot be completely excluded even in products containing iron- or copper-based ingredients. Based on the findings of this preliminary screening, several critical conclusions can be formulated regarding the chemical, technological, and toxicological status of the evaluated products. Quantifiable levels of nickel, copper, and iron confirmed in the analyzed loose formulations are directly governed by the structural and functional composition of the commercial products. Iron functions predominantly as an intentionally incorporated structural colorant rather than an accidental contaminant in the majority of pigmented formulations. Copper exhibits a dual operational status across the evaluated matrix, introducing itself either as a functional skincare active ingredient or occurring as a non-intentional background trace impurity embedded within bulk substrates. In stark contrast, nickel functions exclusively as an unintentional, technologically unavoidable raw-material impurity across all functional categories. The quantitative screening for systemic risks demonstrated that both individual dermal Hazard Quotients and the cumulative Hazard Index across all investigated product categories were multiple orders of magnitude below the commonly accepted toxicological risk assessment benchmark of 1.0. Similarly, the lifetime cancer risk calculated for nickel fell well within safe, negligible parameters below 10^−6^, confirming that chronic dermal exposure to these cosmetic products is highly unlikely to cause systemic toxic or carcinogenic effects to consumers. In stark contrast to the verified systemic safety profiles, the elevated localized concentrations of nickel, reaching up to 37.57 mg/kg, and copper, up to 35.39 mg/kg, determined in the highest-concentration samples pose a distinct risk of triggering localized dermatological reactions. In consumers with pre-existing hypersensitivity or a compromised skin barrier, these absolute concentration burdens are fully sufficient to induce allergic contact dermatitis or peripheral irritant reactions. To successfully bridge the gap between systemic mathematical safety and local sensitization risks, it is recommended that raw material suppliers target a strict technical purity for total nickel in natural mineral cosmetic ingredients.

Ultimately, due to the small sample size of fourteen commercial items and the exclusive dermal-only focus, these results must be interpreted strictly as a preliminary screening rather than a definitive representative map of the entire national market. Nevertheless, they clearly highlight the necessity for continuous, independent surveillance of cosmetic purity and justify the future development of multi-route exposure models that integrate inhalation and ingestion pathways for volatile, loose powder formulations to ensure comprehensive consumer health protection.

## Figures and Tables

**Table 1:** Operating conditions for MP-AES analysis of cosmetic samples.

Element	Wavelength (nm)	Nebulizer Flow (L/min)	Pump Speed (rpm)	Sample Uptake Time (s)	Calibration Fit
Ni	352.454	0.80	15	20	Linear
Cu	327.395	0.75			
Fe	371.993	0.90			

**Table 2:** Selected validation parameters of the MP-AES determination of Fe, Ni, and Cu.

Parameter	Ni	Cu	Fe
Calibration range (mg/L)	0–5	0–5	0–210
Calibration curve fitting, R^2^	0.99997	0.99994	0.99998
LOD (mg/kg)	0.133	0.091	0.199
LOQ (mg/kg)	0.400	0.272	0.597
Repeatability, RSD (%)	8.4 ± 0.7	7.3 ± 2.3	2.6 ± 0.1
Intermediate precision, RSD (%)	9.4 ± 0.9	9.3 ± 2.1	1.8 ± 0.8
Spike recovery, %	97.1 ± 1.1	94.8 ± 2.0	96.1 ± 1.4

LOD—limit of detection; LOQ—limit of quantification; RSD—relative standard deviation; values are presented as mean ± standard deviation.

**Table 3:** Mean concentrations of metals in the analyzed cosmetic samples (mg/kg).

Sample	Ni		Cu		Fe	
	Mean	SD	Mean	SD	Mean	SD
Powders						
P1	1.21 ^d^	0.09	35.39 ^a^	0.30	0.61 × 10^3 ij^	0.03 × 10^3^
P2	6.34 ^b^	0.21	1.80 ^e^	0.11	109.56 × 10^3 a^	2.14 × 10^3^
P3	0.56 ^d^	0.04	4.64 ^cd^	0.30	2.91 × 10^3 gh^	0.12 × 10^3^
P4	37.57 ^a^	0.53	3.02 ^de^	0.11	4.33 × 10^3 g^	0.20 × 10^3^
Bronzer						
BR1	0.45 ^d^	0.02	3.46 ^de^	0.15	10.48 × 10^3 f^	0.34 × 10^3^
Blush						
BL1	0.45 ^d^	0.05	1.03 ^e^	0.04	31.09 × 10^3 c^	0.22 × 10^3^
Highlighters						
H1	3.76 ^c^	0.18	1.40 ^e^	0.21	20.52 × 10^3 d^	0.12 × 10^3^
H2	<LOD	-	0.83 ^e^	0.07	1.99 × 10^3 hi^	0.10 × 10^3^
Eyeshadows						
E1	0.60 ^d^	0.06	1.33 ^e^	0.10	13.16 × 10^3 ef^	0.38 × 10^3^
E2	<LOD	-	2.73 ^de^	0.21	0.03 × 10^3 j^	0.01 × 10^3^
E3	<LOD	-	27.19 ^b^	0.40	15.14 × 10^3 e^	0.07 × 10^3^
E4	0.67 ^d^	0.09	3.43 ^de^	0.23	66.55 × 10^3 b^	0.71 × 10^3^
E5	3.89 ^c^	0.16	2.58 ^de^	0.20	31.41 × 10^3 c^	0.50 × 10^3^
E6	<LOD	-	5.59 ^c^	0.29	0.25 × 10^3 j^	0.02 × 10^3^

SD—standard deviation. LOD—limit of detection (0.133 mg/kg for Ni). P1–P4, face powders; BR1, bronzer; BL1, blush; H1–H2, highlighters; E1–E6, eyeshadows. Means within the same column followed by different superscript letters (a–j) indicate statistically significant differences at *p* < 0.05, evaluated by one-way analysis of variance (ANOVA) followed by Tukey’s post hoc test.

**Table 4:** Non-carcinogenic health risk assessment of the analyzed cosmetic products (CDDE expressed in (mg·kg^−1^·day^−1^); HQ and HI, dimensionless).

Cosmetics		Ni		Cu		Fe		HI
		CDDE	HQ	CDDE	HQ	CDDE	HQ	
Powders	P1	6.83 × 10^−10^	1.26 × 10^−7^	2.00 × 10^−8^	5.00 × 10^−7^	3.47 × 10^−7^	4.96 × 10^−7^	1.12 × 10^−6^
	P2	3.58 × 10^−9^	6.64 × 10^−7^	1.01 × 10^−9^	2.54 × 10^−8^	6.19 × 10^−5^	8.84 × 10^−5^	8.91 × 10^−5^
	P3	3.19 × 10^−10^	5.90 × 10^−8^	2.62 × 10^−9^	6.56 × 10^−8^	1.65 × 10^−6^	2.35 × 10^−6^	2.48 × 10^−6^
	P4	2.12 × 10^−8^	3.93 × 10^−6^	1.71 × 10^−9^	4.27 × 10^−8^	2.45 × 10^−6^	3.49 × 10^−6^	7.47 × 10^−6^
Contouring cosmetics	BR1	1.34 × 10^−10^	2.48 × 10^−8^	1.04 × 10^−9^	2.60 × 10^−8^	3.14 × 10^−6^	4.49 × 10^−6^	4.54 × 10^−6^
	BL1	1.35 × 10^−10^	2.49 × 10^−8^	3.08 × 10^−10^	7.71 × 10^−9^	9.33 × 10^−6^	1.33 × 10^−5^	1.34 × 10^−5^
	H1	7.53 × 10^−10^	1.39 × 10^−7^	2.81 × 10^−10^	7.02 × 10^−9^	4.10 × 10^−6^	5.86 × 10^−6^	6.01 × 10^−6^
	H2	-	-	1.65 × 10^−10^	4.14 × 10^−9^	3.98 × 10^−7^	5.69 × 10^−7^	5.73 × 10^−7^
Eyeshadows	E1	1.44 × 10^−11^	2.67 × 10^−9^	3.20 × 10^−11^	8.01 × 10^−10^	3.16 × 10^−7^	4.51 × 10^−7^	4.55 × 10^−7^
	E2	-	-	6.55 × 10^−11^	1.64 × 10^−9^	7.99 × 10^−10^	1.14 × 10^−9^	2.78 × 10^−9^
	E3	-	-	6.52 × 10^−10^	1.63 × 10^−8^	3.63 × 10^−7^	5.19 × 10^−7^	5.35 × 10^−7^
	E4	1.61 × 10^−11^	2.99 × 10^−9^	8.23 × 10^−11^	2.06 × 10^−9^	1.60 × 10^−6^	2.28 × 10^−6^	2.29 × 10^−6^
	E5	9.35 × 10^−11^	1.73 × 10^−8^	6.20 × 10^−11^	1.55 × 10^−9^	7.54 × 10^−7^	1.08 × 10^−6^	1.10 × 10^−6^
	E6	-	-	1.34 × 10^−10^	3.35 × 10^−9^	5.98 × 10^−9^	8.54 × 10^−9^	1.19 × 10^−8^

CDDE—chronic daily dermal exposure; HQ—Hazard Quotient; HI—Hazard Index. P1–P4, face powders; BR1, bronzer; BL1, blush; H1–H2, highlighters; E1–E6, eyeshadows.

**Table 5:** The lifetime cancer risk (LCR) for nickel in the analyzed cosmetic products (LCR, dimensionless).

Cosmetics		LCR
Powders	P1	2.66 × 10^−10^
	P2	1.40 × 10^−9^
	P3	1.24 × 10^−10^
	P4	8.28 × 10^−9^
Contouring cosmetics	BR1	5.22 × 10^−11^
	BL1	5.25 × 10^−11^
	H1	2.94 × 10^−10^
	H2	-
Eyeshadows	E1	5.63 × 10^−12^
	E2	-
	E3	-
	E4	6.31 × 10^−12^
	E5	3.64 × 10^−11^
	E6	-

P1–P4, face powders; BR1, bronzer; BL1, blush; H1–H2, highlighters; E1–E6, eyeshadows.

## Data Availability

The original contributions presented in this study are included in the article. Further inquiries can be directed to the corresponding author.

## Supplementary Materials

The following supporting information can be downloaded at: https://www.mdpi.com/article/10.3390/toxics14090766/s1, Figure S1: Effect of Fe concentration on Ni and Cu determination by MP-AES: calibration curves for Ni (a) and Cu (b), and relative changes in the emission signals of Ni (c) and Cu (d) at different metal concentrations; Table S1: Comprehensive INCI composition of the evaluated cosmetic products.

## Abbreviations

The following abbreviations are used in this manuscript:

ABS	Dermal absorption fraction
AF	Adherence factor
AT	Averaging time
BW	Body weight
CDDE	Chronic daily dermal exposure
CF	Units conversion factor
CS	Concentration of toxic elements in cosmetics
ED	Exposure duration
EF	Exposure frequency
FDA	Food and Drug Administration
HCA	Hierarchical cluster analysis
HI	Hazard Index
HQ	Hazard Quotient
LCR	Lifetime cancer risk
LOD	Limit of detection
LOQ	Limit of quantification
MP-AES	Microwave plasma–atomic emission spectrometer
PCA	Principal Component Analysis
RfD_d_	Dermal chronic reference dose
RIVM	Dutch National Institute for Public Health and the Environment
RSD	Relative standard deviation
SD	Standard deviation
SF	Carcinogenicity slope factor
