# Supplementary material for: Analysis of Ni, Cu, and Fe in Loose Cosmetic Products: A Preliminary Dermal Exposure and Health Risk Assessment
Source: Toxics. 2026 Aug 27;14(9):766. doi: 10.3390/toxics14090766 (PMC13611473; doi:10.3390/toxics14090766)
Supplement: Supplementary file 1 [file toxics-14-00766-s001.zip › toxics-4446206-supplementary.pdf]

Supplementary materials

Table S1. Comprehensive INCI composition of the evaluated cosmetic products.

Sample Code	INCI Composition List
P1	TALC, ZINC STEARATE, ALUMINUM STARCH OCTENYLSUCCINATE, MAGNESIUM MYRISTATE, CAPRYLYL GLYCOL, MAGNESIUM CARBONATE, PARFUM (FRAGRANCE), ALPHA-ISOMETHYL IONONE, [+/-]: CI 77007, CI 77019, CI 77491, CI 77492, CI 77499, CI 77891
P2	SILICA, POLYMETHYL METHACRYLATE, VINYL DIMETHICONE\ METHICONE SILSESQUIOXANE CROSSPOLYMER, CELLULOSE, VITIS VINIFERA (GRAPE) SEED OIL, AQUA, MAGNESIUM ASPARTATE, ZINC GLUCONATE, SODIUM ASCORBYL PHOSPHATE, TOCOPHEROL, CAPRYLYL GLYCOL, HELIANTHUS ANNUUS SEED OIL, PENTYLENE GLYCOL, CALCIUM GLUCONATE, BUTYLENE GLYCOL, SACCHAROMYCES FERMENT, COPPER GLUCONATE, CHONDRUS CRISPUS EXTRACT, SARCODIOTHECA GAUDICHADII, PHENOXYETHANOL, CI 77492
P3	TALC, MICA, TITANIUM DIOXIDE, HEXYLDECANOL, HEXYLDECYL LAURATE, PETROLATUM, CETYL ALCOHOL, PHENETHYETHANOL, ETHYLHEXYLGLYCERIN, PEG-8, TOCOPHEROL, ASCORBYL PALMITATE, ASCORBIC ACID, CITRIC ACID, MICROCRISTALLINA CERA, CANDELILLA CERA, PARFUM, [+/-: CI 77019, CI 77861, CI 77163, CI 77491, CI 77492, CI 77499, CI 77891]
P4	ORYZA SATIVA (RICE) POWDER, MAGNESIUM STEARATE, PARFUM, PHENOXYETHANOL, ETHYLHEXYLGLYCERIN
BR1	TALC, DIMETHICONE, VINYL DIMETHICONE/METHICONE SILSESQUIOXANE CROSSPOLYMER, BORON NITRIDE, MAGNESIUM STEARATE, SILICA, PARFUM, HDI/TRIMETHYLOL HEXYLLACTONE CROSSPOLYMER, CAPRYLIC/CAPRIC TRIGLYCERIDE, CAPRYLHYDROXAMIC ACID, SALICORNIA HERBACEA EXTRACT, LINALOOL, BENZYL SALICYLATE, ALPHA-ISOMETHYL IONONE, LIMONENE, GERANIOL, COUMARIN, CI 77891, CI 77492, CI 77491, CI 16035, CI 77499
BL1	TALC, PARFUM (FRAGRANCE), BENTONITE, ZEA MAYS (CORN) STARCH, HYDROXYCITRONELLAL, SODIUM METHYLPARABEN, CINNAMYL ALCOHOL, EUGENOL, BENZYL SALICYLATE, LINALOOL, EVERNIA PRUNASTRI (OAKMOSS) EXTRACT, CITRONELLOL, GERANIOL, AMYL CINNAMAL, ALPHA-ISOMETHYL IONONE, HEXYL CINNAMAL, COUMARIN, LIMONENE, BENZYL BENZOATE, BENZYL ALCOHOL, FARNESOL, BENZYL CINNAMATE [+/- (MAY CONTAIN): CI 12085 (RED 36), CI 15850 (RED 6 LAKE), CI 15850 (RED 6), CI 15850 (RED 7 LAKE), CI 15880 (RED 34 LAKE), CI 15985 (YELLOW 6 LAKE), CI 17200 (RED 33 LAKE), CI 19140 (YELLOW 5 LAKE), CI 42090 (BLUE 1 LAKE), CI 45380 (RED 22 LAKE), CI 45410 (RED 28 LAKE), CI 47005 (YELLOW 10 LAKE), CI 73360 (RED 30 LAKE), CI 75470 (CARMINE), CI 77007 (ULTRAMARINES), CI 77163 (BISMUTH OXYCHLORIDE), CI 77491, CI 77492, CI 77499 (IRON OXIDES), CI 77510 (FERRIC FERROCYANIDE), CI 77742 (MANGANESE VIOLET), CI 77891 (TITANIUM DIOXIDE), CI 77019 (MICA)]

H1	MICA, POLYSORBATE 60, ALUMINUM STARCH OCTENYLSUCCINATE, HYDROGENATED POLYISOBUTENE, OCTENYLSUCCINATE, HYDROGENATED POLYISOBUTENE, NYLON-12, TRIDECYL TRIMELLITATE, DIMETHICONE, TRIETHOXYCAPRYLYLSILANE, MAGNESIUM STEARATE, MAGNESIUM ALUMINUM SILICATE, PHOSPHOLIPIDS, BIS-DIGLYCERYL POLYACYLADIPATE-2, GLYCINE SOJA STEROLS, TALC, LECITHIN, POLYHYDROXYSTEARIC ACID, PHENOXYETHANOL, ISONONYL ISONONANOATE, CAPRYLYL GLYCOL, ETHYLHEXYL PALMITATE, ISOPROPYL PALMITATE, ISOSTEARIC ACID, POLYGLYCERYL-3 POLYRICINOLEATE, POLYACRYLAMIDE, AQUA, C13-14 ISOPARAFIN, LAURETH-7. MAY CONTAIN (+/-): CI 77891, CI 77491, CI 16035, CI 75470
H2	MICA, POLYSORBATE 60, ALUMINUM STARCH OCTENYLSUCCINATE, HYDROGENATED POLYISOBUTENE, OCTENYLSUCCINATE, HYDROGENATED POLYISOBUTENE, NYLON-12, TRIDECYL TRIMELLITATE, DIMETHICONE, TRIETHOXYCAPRYLYLSILANE, MAGNESIUM STEARATE, MAGNESIUM ALUMINUM SILICATE, PHOSPHOLIPIDS, BIS-DIGLYCERYL POLYACYLADIPATE-2, GLYCINE SOJA STEROLS, TALC, LECITHIN, POLYHYDROXYSTEARIC ACID, PHENOXYETHANOL, ISONONYL ISONONANOATE, CAPRYLYL GLYCOL, ETHYLHEXYL PALMITATE, ISOPROPYL PALMITATE, ISOSTEARIC ACID, POLYGLYCERYL-3 POLYRICINOLEATE, POLYACRYLAMIDE, AQUA, C13-14 ISOPARAFIN, LAURETH-7. MAY CONTAIN (+/-): CI 77891, CI 77491, CI 16035, CI 75470
E1	MICA, TALC, MAGNESIUM STEARATE, PARAFFINUM LIQIUDUM, ETHYLHEXYL, PALMITATE, POLYBUTENE, DIMETHICONE, METHYLPARABEN, PROPYLPARABEN [+/-: CI 77891, CI 77491, CI 77492, CI 77499, CI 15850, CI 16035, CI 42090, CI 77742, CI 77510, CI 77007, CI 45410, CI 77288]
E2	TALC LAUROYL LYSINE, SYNTHETIC FLUORPHLOGOPITE, ZINC STEARATE, MICA, NYLON-12, HDI/TRIMETHYLOL HEXYLLACTONE CROSSPOLYMER, POLYMETHYL METHACRYLATE, DIMETHICONE, BORON NITRIDE, ISOSTEARYL PALMITATE, ISONONYL ISONONANOATE, CAMELLIA JAPONICA SEED OIL, CAPRYLYL GLYCOL, PHENOXYETHANOL, HEXYLENE GLYCOL, TOCOPHEROL, METHICONE, POLYPERFLUOROMETHYLISOPROPYL ETHER, SILICA, [+/- (MAY CONTAIN): RED 40 LAKE (CI 16035), YELLOW 5 LAKE (CI 19140), BLUE 1 LAKE (CI 42090), CARMINE (CI 75470), ULTRAMARINES (CI 77007), BLACK 2 (CI 77266) [NANO], IRON OXIDES (CI 77491, CI 77492, CI 77499), FERRIC AMMONIUM FERROCYANIDE (CI 77510), MANGANESE VIOLET (CI 77742), TITANIUM DIOXIDE (CI 77891)]
E3	POLYETHYLENE TEREPHTHALATE, DIMETHICONE, POLYBUTENE, VP/HEXADECENE COPOLYMER, DIMETHICONOL, STEARYL DIMETHICONE, POLYURETHANE-11, CI 77510 (FERRIC FERROCYANIDE), CI 16035 (RED 40 LAKE), CI 77000 (ALUMINUM POWDER)
E4	CALCIUM ALUMINUM BOROSILICATE, PENTAERYTHRITYL TETRAISOSTEARATE, SILICA, ISOSTEARYL ISOSTEARATE, DIMETHICONE, CYCLOPENTASILOXANE, TRIETHYLHEXANOIN, CAPRYLIC/CAPRIC TRIGLYCERIDE, POLYSILICONE-11, HYDROGENATED TETRADECENYL/METHYLPENTADECENE, ISODECANE, PHENOXYETHANOL, COLOPHONIUM, ETHYLHEXYLGLYCERIN, MAGNOLIA OFFICINALIS BARK EXTRACT

	[+/-] CI 77891 (TITANIUM DIOXIDE), CI 77499, CI 77491, CI 15850:1, CI 77002, CI 77231, CI 16035:1, CI 77492, TIN OXIDE
E5	SYNTHETIC FLUORPHLOGOPITE, TALC, MICA, POLYETHYLENE, CORN STARCH MODIFIED, SILICA, ISOSTEARYL ISOSTEARATE, CYCLOPENTASILOXANE, ZINC STEARATE, SYNTHETIC WAX, OCTYLDODECANOL, PHENOXYETHANOL, ALUMINUM STARCH OCTENYLSUCCINATE, MAGNESIUM STEARATE, TRIETHOXYCAPRYLYLSILANE, LAUROYL LYSINE, DIMETHICONE/VINYL DIMETHICONE CROSSPOLYMER (AND) SILICA, CAPRYLIC/CAPRIC TRIGLYCERIDE, BENZOTRIAZOLYL DODECYL P-CRESOL, ZINC OXIDE, BUTYLPHTHALIMIDE, ETHYLHEXYL SALICYLATE, BUTYL METHOXYDIBENZOYLMETHANE, KAOLIN, COLOPHONIUM, ETHYLHEXYLGLYCERIN, ISOPROPYLPHTHALIMIDE, HYDROGENATED TETRADECENYL/METHYLPENTADECENE, ISODECANE, POLYMETHYLSILSESQUIOXANE, CYCLOHEXASILOXANE, ISOCETETH-10, MAGNOLIA OFFICINALIS BARK EXTRACT [+/-]: CI 77891 (TITANIUM DIOXIDE), CI 77492, CI 77499, CI 77491, CI 77007, CI 45410, CI 19140, CI 15850, CI 15850:1, CI 77231, CI77002, CI 16035:1, CI 17200
E6	TALC LAUROYL LYSINE, SYNTHETIC FLUORPHLOGOPITE, ZINC STEARATE, MICA, NYLON-12, HDI/TRIMETHYLOL HEXYLLACTONE CROSSPOLYMER, POLYMETHYL METHACRYLATE, DIMETHICONE, BORON NITRIDE, ISOSTEARYL PALMITATE, ISONONYL ISONONANOATE, CAMELLIA JAPONICA SEED OIL, CAPRYLYL GLYCOL, PHENOXYETHANOL, HEXYLENE GLYCOL, TOCOPHEROL, METHICONE, POLYPERFLUOROMETHYLISOPROPYL ETHER, SILICA

P1–P4, face powders; BR1, bronzer; BL1, blush; H1–H2, highlighters; E1–E6, eyeshadows.

Quality Control and Method Validation

Because of the high Fe concentrations in some digest solutions, the potential effect of Fe on Ni and Cu determination was evaluated. Although differences in the slopes of the calibration curves for Ni and Cu prepared in the absence and presence of Fe at 15, 90, and 210 mg/L did not exceed 2% (Figures S1a and S1b), Fe at 210 mg/L, corresponding to its concentration in the BR1 digest, caused relative changes in the emission signals (ΔS, calculated from Equation S1) of approximately 10–12% for Ni and Cu concentrations below 0.1 mg/L (equivalent to 5 mg/kg in the cosmetic sample), as shown in Figures S1c and S1d. Consequently, external calibration was considered adequate for samples with lower Fe contents. For samples with higher Fe concentrations, additional sample dilution or standard-addition calibration was applied.

$$\Delta S = \frac{S_{Fe} - S_0}{S_0} \cdot 100\%, \quad \text{(S1)}$$

where

S_0 is the Ni or Cu signal in solutions without Fe, and S_{Fe} is the corresponding signal in solutions containing Fe.

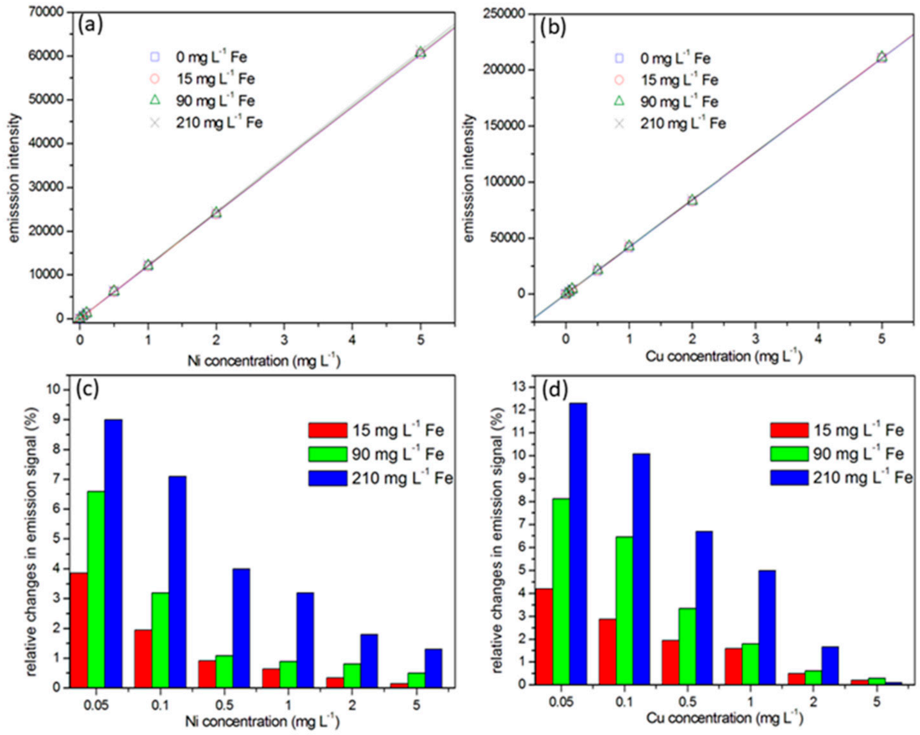

Figure S1. Effect of Fe concentration on Ni and Cu determination by MP-AES: calibration curves for Ni (a) and Cu (b), and relative changes in the emission signals of Ni (c) and Cu (d) at different metal concentrations.

Digestion completeness was evaluated by re-digesting the residue left after the initial digestion cycle, which was visibly present in digests of samples containing silica or silicates. No Fe, Ni, or Cu concentrations exceeding the respective LODs were detected in the solutions obtained after the second digestion step. This confirms the efficient transfer of the investigated elements into solution, including in samples containing substantial amounts of mineral pigments. In contrast, for cosmetics not containing silicon-based compounds, the applied digestion conditions resulted in complete decomposition of the samples.

The robustness of the digestion procedure was assessed by deliberately modifying selected process conditions. Omitting H_2O_2 from the digestion mixture resulted in relative differences in the measured metal concentrations of up to 29% compared with the results obtained using the standard procedure containing 1 mL of H_2O_2. This finding confirms the essential role of the oxidizing agent in the decomposition of the complex cosmetic matrix and the release of the analytes. In contrast, extending the final digestion step to 20 min or increasing its temperature to 210 °C resulted in only minor differences in the measured metal concentrations compared with the standard digestion conditions, with deviations not exceeding 5%. Such differences were regarded as negligible for the purpose of evaluating the robustness of the procedure. Therefore, the developed procedure was tolerant of minor variations in time and temperature conditions; however, the composition of the digestion mixture, particularly the presence of H_2O_2, should be strictly maintained.
